# Structural qualia: a solution to the hard problem of consciousness

**DOI:** 10.3389/fpsyg.2014.00237

**Published:** 2014-03-18

**Authors:** Kristjan Loorits

**Affiliations:** Department of Philosophy, History, Culture and Art Studies, University of HelsinkiHelsinki, Finland

**Keywords:** philosophy of mind, qualia, consciousness, the hard problem, structuralism

## Abstract

The hard problem of consciousness has been often claimed to be unsolvable by the methods of traditional empirical sciences. It has been argued that all the objects of empirical sciences can be fully analyzed in structural terms but that consciousness is (or has) something over and above its structure. However, modern neuroscience has introduced a theoretical framework in which also the apparently non-structural aspects of consciousness, namely the so called qualia or qualitative properties, can be analyzed in structural terms. That framework allows us to see qualia as something compositional with internal structures that fully determine their qualitative nature. Moreover, those internal structures can be identified which certain neural patterns. Thus consciousness as a whole can be seen as a complex neural pattern that misperceives some of its own highly complex structural properties as monadic and qualitative. Such neural pattern is analyzable in fully structural terms and thereby the hard problem is solved.

## INTRODUCTION: THE HARD PROBLEM AS A TENSION BETWEEN THREE THESES

One possible way to present the hard problem of consciousness is to consider three seemingly plausible theses that are in an interesting tension. First, all the objects of physics and other natural sciences can be fully analyzed in terms of structure and relations, or simply, in structural terms. Second, consciousness is (or has) something over and above its structure and relations. Third, the existence and nature of consciousness can be explained in terms of natural sciences. Should the second thesis be incorrect and consciousness fully analyzable in structural terms, then finding the structure of consciousness in some patterns of neural activity (or perhaps in some linguistic-behavioral patterns) and studying the origin and nature of that structure would hopefully reveal us eventually all there is to know about consciousness. On the other hand, if both the first and the second theses are true, it follows directly that consciousness cannot be an object of physics or other natural (or behavioral) sciences and hence its existence cannot be also explained by these sciences.

David Chalmers, the author of the hard problem of consciousness, accepts both the first and the second theses and draws also the conclusion mentioned above. He also adds the premise that what cannot be physically explained is not itself physical (Chalmers, 2003). Therefore he is convinced that the only solution to the hard problem is to endorse some sort of ontological dualism, most preferably a form of property dualism. He argues that traditional natural sciences (for example neuroscience and cognitive science) can perhaps one day explain all the structural-relational properties of consciousness (for example in terms of neural, functional or informational structures and relations), but that consciousness has besides these also phenomenal properties that are in principle out of the reach of traditional scientific methods. However, a number of philosophers have argued that all forms of ontological dualism are philosophically highly problematic, and basically everyone agrees that it would be desirable, if possible, to find a solution to the hard problem without endorsing any form of ontological dualism.

I will argue that the threat of dualism can be avoided and the hard problem can be solved by accepting the first and the third theses while rejecting the second one. In other words, I will argue that the objects of physics and other natural sciences can be indeed fully analyzed in structural terms, but that so can be consciousness. More specifically, I will suggest that the apparently non-structural and monadic elements of consciousness, namely the qualia, are in fact compositional and have an internal structure. According to my proposal, which is based mainly on the work of Francis Crick and Christof Koch (Crick and Koch, 1998; Koch, 2004), the components of qualia are unconscious associations and the structures of qualia are the structures of networks of these unconscious associations. I will argue that those structures can be also described in neural terms and thereby identified with certain neural patterns. Shortly, according to my view, qualia can be analyzed in fully structural terms and identified with certain neural patterns.

Since the formulation of the hard problem I am using (the formulation, according to which it is the tension between the three above presented theses) is not a typical one, perhaps a few words should be said about it before proceeding.

There are two main reasons for me to favor the above presented formulation: the first one is rhetorical and the second is strategic. The rhetorical reason is the following: the formulation I have chosen summarizes nicely some central ideas about the hardness of the hard problem. Namely, most presentations of the hard problem include the idea according to which all the so called easy problems of consciousness are “easy” because they are problems of explaining some *functions* of consciousness. The hard problem is, accordingly, a problem of the existence of certain properties or aspects of consciousness which cannot be analyzed in terms of functions. Similarly, Chalmers (2003) rejects physicalism on the grounds that every physical phenomenon can be analyzed in terms of structure and dynamics, but that consciousness has certain properties or aspects which cannot be analyzed in such terms. It has been also argued that empirical methods have access only to dispositional properties, but that consciousness has besides those also properties that cannot be analyzed in terms of dispositions. The list of similar arguments could be continued. What is common in all of them is the idea that every object of natural sciences can be analyzed in terms of some structures (causal, dispositional, functional, spatiotemporal, relational, informational, etc.), but that certain properties or aspects of consciousness cannot.

I am aware that a position according to which every object of natural sciences can be analyzed in terms of some *specific* type of structures is strictly speaking not the same one as the position according to which every object of natural sciences can be analyzed in structural terms *tout court*. I am also aware that the ideas described above are typically used as parts of the arguments against physicalism and not as formulations of the hard problem itself. If someone wants to reject my formulation on those grounds, she is welcomed to do so. For my purposes it is actually enough to recognize the problem I have formulated as a philosophical problem that is related to the hard problem in a simple and straightforward way which I will specify below.

The most common ways to introduce the hard problem are intuitively appealing but rather obscure in meaning. So, the hard problem is typically introduced as the problem of explaining how the conscious experience “rises” from neural activity or why is there something “it is like to be” conscious. An important phase in every careful presentation of the hard problem is therefore specifying the meanings of the obscure expressions used in those intuitively appealing introductions. While talking about unique and philosophically significant features of conscious states (Chalmers (2003, p. 103) writes: “Each of these [conscious] states has a phenomenal character, with phenomenal properties (or qualia) characterizing what it is like to be in the state.” Then he specifies the meaning of “qualia” in a following endnote (p. 135): “On my usage, qualia are simply those properties that characterize conscious states according to what it is like to have them.” So, in the context of the hard problem, qualia or phenomenal properties are exactly those properties or aspects of consciousness whose existence seems to be inexplicable in the framework of traditional natural sciences. Therefore, the most generally put, the essence of the hard problem is that some properties or aspects of consciousness (however we decide to call them) appear to be inexplicable in the framework of traditional natural sciences.

Now I am ready to state my strategic reason for using the formulation presented above: by formulating the hard problem as a tension between the three theses presented above and then approaching it by using the strategy mentioned earlier (arguing that the objects of physics and other natural sciences can be indeed fully analyzed in terms of structure and relations, but that so can be consciousness) I hope to demonstrate that there are no such properties or aspects of consciousness that cannot be explained in the framework of traditional natural sciences. Therefore I will proceed, for the sake of simplicity, as if my formulation is a legitimate formulation of the hard problem. Anybody who feels that it is in fact not, can consider it as a formulation of a separate problem which is related to the hard problem in the following straightforward way: solving the problem I have formulated by using the strategy I am using solves also the hard problem. And this is all that should matter at the end of the day.

To sum up, my strategy is based on a simple idea, a conditional, which I believe to be undeniable: *If* a phenomenon is analyzable in *fully* structural terms, then explaining the origin and nature of the structure of that phenomenon amounts to explaining the origin and nature of the phenomenon itself. I will argue that we have a good reason to believe that consciousness is in fact analyzable in fully structural terms and that contemporary neuroscience can offer us a partly speculative, but nevertheless plausible idea about the nature and origin of that structural phenomenon.

## SCIENTIFIC OBJECT STRUCTURALISM

Before proceeding to the most important part of my argument, which is the rejection of the second thesis by offering a fully structural account of consciousness, I will consider briefly why we should accept the first thesis. Firstly, the position that stresses the structural nature of objects of natural sciences is very widely accepted. Let us call it *scientific object structuralism*. It is held by Lockwood (1989), Strawson (2006), Stoljar (2001), Mermin (1998), Eddington (1928), Chalmers (1996), Russell (1927), Seager (2006), Shoemaker (1994), Ladyman and Ross (2007), to mention only few. Among numerous supporters of very different forms of scientific object structuralism there are both defenders and critics of ontological dualism, and moreover, even though the position is often presented in the context of philosophy of mind (for example Russell, 1927; Chalmers, 1996; Seager, 2006), it has been also frequently put forward in a much broader contexts of philosophy of science and metaphysics (for example Shoemaker, 1994; Ladyman and Ross, 2007).

Put informally, the main idea of scientific object structuralism is that every piece of relata in whichever network of relations studied by natural sciences can be analyzed further in relational terms. Also, almost every element of whichever structure can be arguably analyzed in terms of some finer-grain structure. And supposing there are some fundamental elements with no finer-grain internal structure, it would be still arguably true that these elements are empirically accessible only via their (causal) relations with other elements and objects (including, perhaps, some measuring apparatus). In other words, all our knowledge about them is limited to the relations they have with other objects (and eventually to us).

However, only a relatively small minority of the proponents of scientific object structuralism believe that structure and relations are actually all there is. An example of that kind of radical structuralist metaphysics is a theory of Ladyman and Ross (2007). According to their position, known as ontic structural realism (also as radical structuralism), pieces of relata and elements of structures in theories of natural sciences are merely heuristic devices with no fundamental ontological status. Shoemaker (1994) argues in a similar spirit that causal relations and causal structures are the only thing ontologically fundamental. His position is sometimes referred to as causal essentialism.

Many scientific object structuralists defend a less radical position, known as epistemic structural realism, according to which structure and relations are simply all we can empirically access. Some proponents of epistemic structural realism argue that even though we cannot have any empirical evidence for the existence of non-structural fundamental relata, we have to assume their existence in order to make sense of the idea of there being any relations in the first place. In other words, they argue that there could be no relations without some fundamental relata (for example Esfeld, 2004).

Some philosophers defend yet weaker form of scientific object structuralism. According to them it is true that traditional scientific methods have no access to anything but structure and relations, but that the existence of something over and above structure and relations can nevertheless be perceived. Namely, they hold that the existence of our immediate conscious experience is known to us directly and that we can also “see” that our consciousness is something over and above its structure – it is arguably something that *has* a structure, not something that merely *is* a structure. From the group of philosophers mentioned above the proponents of that position are Chalmers (1996), Russell (1927), Seager (2006).

In addition to the fact that the core idea of scientific object structuralism is very widely accepted, it is also hard to see how rejecting it could help us solve the hard problem of consciousness. The only way I could imagine this to happen, is if someone demonstrated that at least some of the perfectly ordinary objects of natural sciences have such irreducibly non-structural properties whose existence can be experimentally verified and is also philosophically unproblematic. Namely, if the existence of such properties was not experimentally verified but simply assumed in result of some philosophical considerations, then those properties would not be proper objects of natural sciences after all. And if their existence was philosophically problematic, it would not solve the hard problem but simply expand it to some other phenomena besides consciousness. And as far as I know, no one has yet demonstrated that any ordinary objects of natural sciences have (or could have) such properties.

On the other hand, the idea that consciousness has some features over and above its structural and relational properties has much less supporters and has in fact been strongly criticized by many (for example by most of the functionalists, behaviorists, and representationalists). However, most of the attempts to analyze consciousness in fully structural terms have ended up eliminating or simply ignoring certain (qualitative) aspects of consciousness whose existence is considered as absolutely obvious by many. In other words, it has been hard to come by with a theory of consciousness that would satisfy both structuralists and qualia realists. Below I will try to sketch a framework which, I believe, should appease both parties.

## STRUCTURE OF CONSCIOUSNESS AND QUALIA

Proponents of the non-structural view of consciousness have often suggested that the non-structural elements of consciousness are the so called qualia – supposedly monadic and qualitative features of conscious experience. Qualia are typically considered to be private to the one experiencing them and ineffable by nature. The paradigmatic examples of qualia are simple color experiences or raw feels: the redness of red or the painfulness of pain. So, the typical framework behind the non-structural view about consciousness would look something like this: Substantial building blocks of consciousness, namely the qualia, are connected by numerous complex relations and forming numerous complex structures. An individual consciousness as a whole would be hence some kind of structured bundle of qualia. Arguably, the structure of such bundle could in principle turn out to be identical with a structure of a certain pattern of neural activity, which would be in principle accessible by methods of future neuroscience (even Chalmers, 1995, 2003, believes that the structure of consciousness is identical with some informational structure in our brains), but the qualitative properties of qualia could not.

The question of what does it exactly mean that the structure of consciousness could turn out to be a structure of a neural activity pattern is obviously a tricky one. An impressive attempt to answer it is made by Revonsuo (2006). He believes that the structure of consciousness will be found in the brains once we discover and learn to monitor the *proper level of organization* of the neural activity – in any other level we would find only the *neural correlates* of consciousness (NCC). In other words, in those lower levels we would find some patterns of neural activity that correlate with the content of our consciousness, but we would not understand why those correlations occur and what their nature is. On the proper level of organization, on the other hand, we would find a pattern that simply *has* a structure consciousness. However, (Ladyman and Ross (2007, pp. 53–57) criticize strongly the idea of there being different ontological levels in nature because, according to them, there are plenty of natural phenomena which do not fit nicely to the framework of hierarchically organized levels. Nevertheless, they are not denying the obvious fact that structural patterns in nature are often organized in a semi-hierarchical manner. Fortunately, the main ideas of Revonsuo (2006) seem to be easily conveyable from the framework of levels to the more flexible one allowed by Ladyman and Ross (2007), so that the structure of consciousness could be seen as a pattern of certain other patterns of certain yet other patterns etc. of some simple neural events.

Still, according to the view of Chalmers and others who believe that qualia are irreducibly qualitative, even if we could get from mere unexplained correlations between some neural activity pattern and consciousness to the detailed structural identity (or structural isomorphism) between the two, we could still not establish full identity between them because qualia would be essentially non-structural and could not be, therefore, identified with any structures. For example, in case of visual consciousness, one could argue that even if we could one day “see” by scanning someone’s brain that she has a visual experience of a red apple on a green plate (and even if we could detect all the structural details of the perceived scene), we would still (arguably) have no idea why the redness of the red and the greenness of the green are experienced by her the way they are and not, for example, other way around (and moreover, why are they experienced in such a vivid and qualitative fashion at all).

The supposed privacy and ineffability of qualia has made theories about them vulnerable to philosophical arguments based on the largely supported view that the nature of language and meaning is essentially public and intersubjective. It has been often argued in Wittgensteinian or Quinean fashion that the concept of private object is philosophically highly problematic because absolutely private objects could have no role in language or in any of our theories. Generally, the Wittgensteinian attitude toward consciousness tends to lead to an externalist view about the phenomenon: it seems that if all our references about the content of our consciousness are actually made by using the vocabulary of extramental public objects, then the proper theory of consciousness should be a theory about our linguistic-behavioral interactions with the extramental world (see, for example, Lagerspetz, 2002). However, there seems to be a philosophically rather shallow point of view from which it makes perfect sense to claim that a mental content can be private (but not private in the philosophically problematic absolute sense): namely, a neurobiological point of view, from which people can be seen as biological cognitive systems with limited communicative skills.

There seems to be no deep philosophical mystery about an idea of a cognitive system that has certain information about some of its inner states, but lacks the ability to communicate that information to others. An excellent example of that kind of neurobiological description of human beings has been put forward by Crick and Koch (1998), Koch (2004). Besides explaining the (non-absolute) privacy of qualia, Crick and Koch also offer an excellent account of the apparently monadic nature of qualia.

Before proceeding, it should be noticed that even though I build my case here solely on the theory of Crick and Koch (and also “fine-tune” my arguments accordingly), the general strategy I am using is compatible with whichever (neurobiological) fully structural account of qualia. For instance, there are several theories according to which individual qualia are defined by their location in the complex multidimensional qualia space (or simply, by their similarity and dissimilarity relations with other qualia), for example, Churchland (1986), O’Brien and Opie (1999), Edelman (2003), Pestana (2005). Those theories could be, in principle, interpreted so that the structure of an individual quale is the structure of the network of all the similarity and dissimilarity relations the quale has with other qualia. Similarly, according to Balduzzi and Tononi (2009), each individual quale is a certain “shape” in a qualia space – a shape that embodies certain set of informational relationships. So, the structure of such set of relationships could be seen as a structure of the corresponding quale.

The main reason why I have chosen to focus on the theory of Crick and Koch is that I find their approach intuitively particularly appealing, for it allows us to understand the hypothetical structural nature of an individual quale both in neural and in phenomenal terms.

Let us consider someone’s conscious experience of the color red. According to Crick and Koch, the structure of such reddish color experience (or the *meaning* of that experience) is a vast network of unconscious associations of all the countless encounters with red objects in that person’s personal history and of personal histories of her ancestors, embodied in her genes (Crick and Koch, 1998; Koch, 2004, pp. 242–244). The peculiar phrase “embodied in her genes” means simply that not all the unconscious associations are formed during a person’s lifetime as a result of her interaction with the environment, but that some of them are innate: programmed by the evolution, so to speak.

Crick and Koch (1998) also manage to give an account of these associations in terms of neural processes. According to them, there is an *explicit neural representation* for every aspect of our conscious experience. By an explicit neural representation they mean an increased activity of a “smallish group of neurons” (most likely between 100 and 1000) situated close together. Those groups of neurons can be also called *essential nodes* (Koch, 2004, pp. 34–35). Every time when the activity of one of such essential nodes is above certain threshold, a person is conscious of the corresponding aspect (it could be a color, a shape, a direction of movement, a familiar object, etc.).

In order to avoid various philosophical problems related to the difficult concept of neural representation (see, for example, Hutto and Myin, 2013), those neural events should not be seen as representations *in themselves* in any deeper metaphysical sense. The fact that the increased activity in certain essential nodes systematically co-occurs (in proper conditions) with the typical (verbal) reactions to certain aspect of consciousness (for example, a subject reporting of seeing something red) is the only *prima facie* reason for us to call the activity of these essential nodes explicit neural representations. One of the main reasons for such systematic co-occurrences is, according to the hypothesis, the fact that all the essential nodes responsible for explicit representations are directly connected to the planning modules of the brain (the prefrontal and anterior cingulate cortices, in particular), where their projections can easily affect the behavior of the subject (Koch, 2004, p. 245).

Therefore, according to the hypothesis, the totality of all the explicit neural representations has a detailed and exact correlation with the content of the person’s consciousness. Since all the essential nodes responsible for the explicit neural representations are (by the hypothesis) also connected to the planning modules of the brain, it means that the functional structure of the whole network of the explicit neural representations would actually be the functional structure of the corresponding consciousness. In other words, the causal effects of the network described above are supposedly identical to the causal effects of our consciousness (that is why we can report most of the aspects of the content of our consciousness).

However, the question about the nature of qualia remains: why should the increased activity in an essential node have a specific, yet ineffable qualitative feel? According to a somewhat speculative hypothesis of Crick and Koch, the quale associated to an explicit neural representation is the *meaning* of that representation to the rest of the brain. In psychological and phenomenal terms that meaning is, as mentioned before, a vast network of various unconscious associations. In neural terms it is the network of all those neural connections that the essential node in question has with other essential nodes.

When the activity of some essential node rises above required threshold for the corresponding aspect to become a part of consciousness, then the activity of most of the connected essential nodes rises slightly, but stays below the required threshold. However, the slightly increased activity of the vast network of all the connected essential nodes is collectively strong enough to affect person’s attitude toward the consciously experienced aspect. Then the person becomes conscious of the corresponding aspect and its rich and specific meaning (the quale), but stays ignorant of the single unconscious associations composing that meaning (the components and structure of the quale).

In order to understand the situation in phenomenal terms it would be perhaps better to think of the so called unconscious associations not as absolutely unconscious, but as vaguely conscious (perhaps as a “tip of a tongue” kind of conscious – conscious without a quale but with an ability to recognize the missing quale instantly, should it pop up, as “the right one”). So, the slightly increased activity of any single essential node that corresponds to some vaguely conscious aspect would be too week to cause any significant activity in the planning modules of the brain, and therefore the subject could not report any conscious experience of the corresponding aspect. However, the slightly increased activity of the vast network of all the connected essential nodes that corresponds to a quale would be collectively strong enough to cause some neural activity in the planning modules, and so the subject could report of experiencing something peculiarly specific, but she would not be able to distinguish or recognize (or report) any single vaguely conscious components of that experience.

Since different networks of unconscious (or vaguely conscious) associations would have different influences on the planning modules, the person could identify different networks of unconscious associations without having conscious access to their structures. That is why these networks would appear to her monadic and their differences qualitative (even though they are in fact highly complex and in principle analyzable in structural terms). Since the person would have no conscious access to the complex structure of her qualia, she could not obviously communicate it to the others. So, in this sense, qualia would be truly private and ineffable to the one experiencing them.

By analogy, we can consider some macro-physical properties of an ordinary physical object made of wood and stone: if we examine such object at a low enough level of detail, we can call the macro-physical properties of woody and stony qualitative properties (here I use the word “qualitative” in a strictly physical sense, as, for example, wood and stone have different qualities from the perspective of a construction engineer or an architect). In order to understand the qualitative difference between woody and stony in terms of the internal structures of those materials, we would have to enter some finer-grained level – for example to the one in which we find the structures of single molecules. In case of consciousness we are simply dealing with a cognitive system that is not capable of examining its own inner structure at the level where the qualitative properties of qualia are analyzable in structural terms.

Since the hypothesis presented above contains an idea according to which people are ignorant of the fundamental (structural) nature of their qualia, it has some superficial resemblance to the so called *epistemic view* or *ignorance hypothesis*, put forward by Stoljar (2006). To avoid confusion, it should be recognized that the main idea and strategy of Crick and Koch are actually very different from the one of Stoljar. The main idea of Stoljar is, in a nutshell, that we are *scientifically ignorant* about the nature of consciousness and that this is why we fail to see how consciousness could be reducible to anything physical (or non-experiential, as Stoljar puts it). It is clear that the philosophically relevant ignorance in the theory of Crick and Koch is not scientific ignorance, but an ignorance of individual human beings. The ignorance of individual human beings is part of their cognitive architecture and there is no reason why we could not have scientific knowledge about that architecture. For example, when I have a visual perception of a red apple, I have a direct epistemic access to many structural features of my visual experience: the size and shape of the perceived apple, for instance. I do not have similar direct epistemic access to the structure of the perceived redness of my visual experience, but this does not mean that I could not be a member of a scientific community that has scientific knowledge about that structure.

Another philosophical view that has deeper and more substantial resemblance to the theory of Crick and Koch is the so called *introspective inaccuracy hypotheses*, put forward by Pereboom (2011). According to (Pereboom (2011, p. 14) it is a serious open possibility that the introspective mode of presentation misrepresents the qualitative nature of qualia (or phenomenal properties). (Pereboom (2011, pp. 16–17) also suggests that the nature of that misrepresentation could be such that the qualia (or phenomenal properties) are actually compositional and complex, but appear in introspection as primitive and monadic. If Crick and Koch are right, then qualia are indeed compositional and complex, even though they appear to us as primitive and monadic. Therefore, if Crick and Koch are right, then the introspective inaccuracy is in fact much more than just a serious open possibility: it is an actual matter of fact. Therefore it is interesting that Pereboom does not mention the work of Crick and Koch or any other neurobiological structural account of qualia. I am not going to speculate why he does not, but I think it is important to notice that even though the views of Pereboom and Crick and Koch are, as far as I can tell, fully compatible, the theory of Crick and Koch is much more experimental and naturalistic in spirit and much less philosophical. On the other hand, since Pereboom offers a detailed analysis of how the introspective inaccuracy hypotheses would answer the most important and famous philosophical arguments related to the hard problem (the so called conceivability argument and knowledge argument), his treatment might be considered to add a significant philosophical credibility to the hypothesis of Crick and Koch.

One seemingly substantial difference between the views of Pereboom and Crick and Koch is that (Pereboom(2011, p. 14) suggests that phenomenal properties might not actually have any qualitative nature, while Crick and Koch are explicitly realists about qualia and their qualitative nature while denying simply that qualia are *fundamentally* qualitative. I suspect that the above difference is not in fact substantial at all, but merely terminological: Crick and Koch are realists about the qualitative nature of qualia in the sense that one can be realist about macro-physical qualitative properties of ordinary physical objects, for example the woodiness of wood or the (physical) redness of a red tomato, while recognizing that such macro-physical qualitative properties are not in fact fundamental, but analyzable in fully structural terms in some finer-grained level. Pereboom, on the other hand, questions the qualitative nature of phenomenal properties in the sense that one can question the existence of qualitative macro-physical properties of ordinary physical objects on the grounds that these properties are actually not fundamentally qualitative, but analyzable in structural terms in some finer-grained level. So it seems that the two views are actually in a substantial agreement, but I prefer qualia-realistic terminology of Crick and Koch for rhetorical reasons: we are so impressed by the undeniable and vivid presence of the qualia because qualia do exist and we have an immediate and direct cognitive access to them, although we do not have an immediate and direct cognitive access to their internal structures.

To sum up, according to the framework introduced by Crick and Koch, qualia are highly complex and perfectly public structural-relational properties of some cognitive systems, even though those systems themselves perceive them as monadic and private. The blueness of blue and the redness of red are qualitatively different because the structures of the networks of their composing unconscious associations are different. Similarly, the quality of a red quale feels exactly the way it does because the structure of the network of its composing unconscious associations is exactly such as it is.

Of course, a skeptic would not be convinced: so far I have simply asked the reader to believe that the specific quality of a quale is a result of its internal structure, but unless there is a way it could be somehow phenomenally verified, we have no compelling reason to believe so. Therefore I will consider next a situation that could be, in my opinion, interpreted as having a direct glimpse at the internal structure of an apparently monadic quale.

Daniel Dennett offers an example of a situation where an apparently monadic quale is analyzed phenomenally into several components: the quale of a low guitar sound seems to be monadic at first, but will be perceived as an ensemble of many sounds after we have isolated and listened the individual overtones that compose the original sound (Dennett, 1991, pp. 49–50). Dennett, who is famously known for denying the existence of qualia, uses the above example to demonstrate how confused we are about the nature of our sensory perceptions. However, the framework of Crick and Koch allows us interpreting Dennett’s example as a situation in which a small part of the unconscious structure (the overtone structure) of an auditory quale becomes conscious. What is interesting is that once a person has learned to recognize the individual overtones of the sound, she also, in a sense, understands why the ensemble of these overtones sounds the way it does. In other words, most of the people would be, in the situation described above, intuitively willing to admit that the overtone structure more or less determines the guitarish quality of the composed sound: they would still hear the original sound, but not as a monadic and ineffable quale but as the ensemble of its overtones. Also, almost everyone would agree that the composed sound is somehow phenomenally richer than any of its individual overtones and that this richness can be perceived as well before as after one learns to hear the overtones in the composed sound. It is as if we could somehow perceive that there is a lot of information in some apparently monadic quale, but could not tell what kind of information that is. Once we become aware of the overtone structure, we get access to some (a tiny part) of that information.

However, it should be noticed that above exercise would not allow us to leave the space of qualitative experience, for all the experienced individual overtones would have qualia of their own. Nevertheless, the exercise would allow us to see (assuming it has been fully successful) that the auditory quale, which we used to believe to be as monadic and ineffable as phenomenal redness, has actually an internal structure that more or less determines its specific phenomenal character.

Of course, it could be wondered if the mere feeling (or an intuition) that the perceived guitarishness is compositional allows us to conclude that it is *actually* compositional. Fortunately, in this particular case the mere feeling (or an intuition) seems to be all the evidence we need. Namely, the only reason for us to believe that the phenomenal guitarishness was monadic and not compositional, was that we felt it was monadic and not compositional. And once that feeling is removed by the exercise described above, the corresponding belief should be abandoned as well.

The above example is important, for it helps us understand intuitively why our qualia have so peculiarly specific natures. In other words, it helps us understand why different qualia are not simply characterized by apparently ineffable “somethingness,” but have each a very specific apparently ineffable “suchness.” For example, should we train our ear to distinguish between the overtone structures of the sounds of guitar and trumpet, we would, supposedly, understand intuitively (or “perceive directly”) what are the structural natures of the qualities like “guitarish” and “trumpetish” and why each of them has its specific qualitative “suchness.”

From here we could continue with wilder speculations and imagine a technique or a device that could help us become conscious of some associations that are essential components of our color qualia. Perhaps such device could locate the essential nodes that correspond to the most important unconscious associations of some quale (the ones with the strongest influence on the planning modules). After that the device could stimulate those nodes and turn the corresponding unconscious associations conscious. Then, supposedly, we would understand intuitively why the redness of the red quale and the greenness of the green quale appear to us the way they do and not the other way around. It is important to notice that if the theory of Crick and Koch is approximately true, then the above speculation is not merely a thought experiment but an empirical prediction – an actual experiment for the future scientists to design. The similar examples can be put forward about smells, tastes or moods. For example, it is traditionally held that certain unconscious memories of some traumatic events can *cause* depression or anxiety, but perhaps the better way to think of the situation would be, in the light of the theory of Crick and Koch, that those unconscious memories are *parts* or *components* of the depression or the anxiety.

One could still argue that the problem remains, for the consciously experienced components of guitarishness and blueness would have qualitative properties of their own. But according to the hypothesis, (most of) those components would be initially unconscious and “qualialess” – they would acquire qualia only at the moment they become conscious (when the activity of the corresponding essential nodes reaches the threshold and activates their own networks of unconscious associations). And then, as predicted by the hypothesis and suggested by Dennett’s example, a subject would recognize them as components of the original quale and also realize that she was not conscious of them before. Nevertheless, because of the enormous structural complexity of qualia, the subject would never become directly conscious of the full structure of her quale.

So, the way I see it, the present evidence for the hypothesis according to which qialia are fully structural comes mainly from neurobiology. But in the future the hypothesis could be significantly strengthened by the evidence from phenomenal experience. Namely, the hypothesis predicts that whichever apparently monadic and non-structural quale we choose to pick, with the help of our future device, it would reveal its structural (even though not fully structural) nature. And that makes the hypothesis empirically falsifiable: should we find a quale that could not be phenomenally decomposed by our hypothetical future device, we would have a concrete evidence against the compositional theory of qualia presented here.

It is true that the theory of Crick and Koch is partly speculative and could be wrong in many of its details. However, their theory gives us a positive and rather specific idea of how the existence and nature of qualia could be explained in structural terms, and hence it offers us a good and scientifically inspired reason to believe that the apparently monadic and non-structural character of qualia is in fact not fundamentally qualitative and monadic. My main goal is not to use the hypothesis of Crick and Koch for developing another philosophical argument for the possibility of structural analysis of qualia, but to present their neurobiological theory as an actual hypothetical structural description of qualia: a description which is very coarse and by large part speculative, but which would solve the hard problem because it could be understood both in neural and in phenomenal terms. In other words, my aim is to present the theory of Crick and Koch as a coarse and hypothetical but fully structural description of a natural phenomenon that would be recognized at the same time as a description of phenomenal consciousness and a description of certain neural activity. The description of phenomenal consciousness is recognized, when the structure is described in terms of unconscious associations and illustrated with examples like the one borrowed form Dennett. The description of neural activity, on the other hand, is recognized, when the *very same structure* is described in terms of explicit representations and essential nodes. In other words, according to the hypothesis, the relational structure of the whole network of the active essential nodes (both the ones corresponding to aspects of consciousness and the ones corresponding to unconscious associations) is identical to the relational structure of consciousness (including qualia).

This is where I would like to rest my case. A fully structural account (in the sense that it does not contain any irreducibly non-structural elements) of consciousness and qualia together with a speculative, but plausible theory of how such structure is actually (identical to) the structure of a certain neural activity pattern is, in my understanding, nothing less than a solution to the hard problem. It answers the question of how phenomenal consciousness could possible “rise” from neural activity: if the hypothesis is correct, then the phenomenal consciousness simply *is* a certain complex pattern of neural activity: a pattern of patterns of patterns etc. of some simple neural events. It also answers the question of why is there something “it is like to be” conscious: if “qualia are simply those properties that characterize conscious states according to what it is like to have them,” as (Chalmers (2003, p. 135) puts it, then neuroscientifically intelligible structural account of qualia is also neuroscientifically intelligible structural account of why there is something it is like to be conscious. In other words, the question of why is there *something it is like to be* conscious is, according to Chalmers, the question of why qualia exist. And the main reason why we are scientifically more puzzled by the existence of qualia than, for example, by the existence of hydrogen atoms, chairs or neural processes, is that in the case of the latter we could easily understand how they are analyzable in fully structural terms (even though we might not have such an analysis ready at hand), but in the case of qualia we cannot. But once we succeed in analyzing qualia in fully structural terms and identifying those structures with certain neural activity patterns, the question of why qualia exist can be seen as a question of why those neural activity patterns exist. And that question could be, hopefully, eventually answered by the combined efforts of neurobiology, evolutionary neuroscience, cognitive science and possibly some other empirical disciplines.

And last but not least, the above structural account of consciousness is psychologically convincing and intuitively illuminating: it is much easier to accept (for me, for Crick and Koch and, hopefully, for many others) that the constitutive components of qualia are unconscious associations, than, say, some fundamental “protophenomenal” elements of whose nature we are completely ignorant. Of course, it should be recognized that the view I have proposed here is far from forced upon us by the evidence. Namely, since we could, according to the hypothesis, actually never experience directly the full structure of any of our qualia, we could also never establish the identify between qualia and certain neural activity patterns with the same certainty we can establish identity between the macro-physical quality of woodiness and some microphysical properties of wood. Nevertheless, we could understand how qualia *could* be identical with certain neural activity patterns and how we could gather (both neurobiological and phenomenal) evidence to support the idea of such identity.

## SOME PHILOSOPHICAL CONSIDERATIONS

Finally, I would like to consider how the framework I have suggested relates to some of the well-known arguments and thought experiments used to illustrate the problematic hardness of the hard problem. As mentioned earlier, the approach of Crick and Koch is very naturalistic and rather unphilosophical. As (Koch (2004, p. 316) himself puts it: “You can’t reason your way to an explanation of consciousness. Brains are too complicated, and are conditioned on too many random events and accidents of evolutionary history, for such armchair methods to successfully illuminate the truth.” Such empirical spirit should be seen as a particular strength of their approach: it seems to be much more preferable to come by with an actual (even though hypothetical) scientific account of consciousness and qualia than to simply demonstrate philosophically that such scientific account could be developed. Should such scientific account be successful, then the philosophical arguments against (as well as for) its possibility would lose most of their appeal. Therefore I do not attempt to put forward any fully developed arguments for the philosophical plausibility of the hypothesis of Crick and Koch in this section, but merely to consider the possible nature of the impact of their theory, should it be approximately true, to the philosophical debate about the issue. Anyhow, since the theory of Crick and Koch is, as far as I can tell, philosophically fully compatible with the introspective inaccuracy hypotheses, put forward by Pereboom (2011), one can look for more detailed and technical approach to the philosophical problems considered below from his book.

It has been often argued that it is *ideally positively conceivable* that a creature physically identical to some conscious human being (for example to you or to me) could nevertheless lack qualia, in other words, that it could be some sort of unconscious zombie. Similarly, it has been claimed that it is ideally positively conceivable that someone physically and functionally identical to you or to me could have his or her qualia inverted: for example, in situations where I would experience the red quale, he or she would experience the green quale and vice versa. Arguably, there would be no way of telling if someone’s qualia are inverted, for there would be no physical or functional signs of it. (It has been argued that from the above described conceivability would follow many important metaphysical facts, including the non-physical nature of consciousness. Since I am denying the ideal positive conceivability of zombies and inverted qualia, I will not examine any of such arguments here.)

It seems rather obvious that if qualia can be analyzed fully in structural terms (as networks of unconscious associations) and if the structures of qualia are implemented by some patterns of neural activity, then any creature that is physically identical to a conscious human being would also have the exact same qualia as she does. Namely, it would be logically inconsistent to hold that some *fully* structural phenomenon could be somehow different or even absent in an occasion where its structure is present. Therefore, a zombie would be no more conceivable than a physical object that has the exact molecular structure of wood, but lacks nevertheless the physical quality of woodiness. Likewise, the inverted qualia would be no more conceivable than a sound that has the overtone structure of a guitar sound, but sounds nevertheless trumpetish. Shortly, zombies and inverted qualia would not be ideally positively conceivable.

It has been also argued that there is an unbridgeable epistemic gap between neural activity and qualia. From the existence of such gap it has been inferred, among other things, that consciousness cannot be fully analyzed in neural terms. It is often claimed, by referring to the famous article of Nagel (1974), that any amount of objective knowledge about, say, a bat’s brain can never contain the knowledge of *what is it like to be* a bat (in other words, what is the exact qualitative character of a bat’s consciousness). (Nagel (1974, p. 440) himself also claims that “[t]he subjective character of the experience of a person deaf and blind from birth is not accessible to me, for example, nor presumably is mine to him.” Similarly, Jackson (1986) argues (and many agree) that a person who spends all her life in a black-and-white environment, even if she is a skilled and well educated neuroscientist, could never know what is it like to see red.

According to the framework I have suggested, based on the neurobiological theory of Crick and Koch, the subjective and qualitative characters of the consciousnesses of bats, blind persons, persons raised in black-and-white environments etc. can be all described in structural terms, even though the above creatures themselves would fail to do so, as individuals, in respect to their own consciousnesses. Therefore, in a sense, there truly is an epistemic gap, but it should not be thought of as a necessary gap in our scientific knowledge, for it is always a gap in some particular cognitive system’s individual knowledge. In some cases we can imagine how such gap could be bridged with the help of some hypothetical futuristic technology. For example, if we could alter in a proper way the neural structure of a blind person or a person who is raised in a black-and-white environment, we could in principle convey them the knowledge of what is it like so see or what is it like to see red. However, the case of us not knowing what is it like to be a bat seems to be difficult because the cognitive structures of bats and people are simply too different. Even if we could turn a person’s neural structure into a neural structure of a bat, we would have simply turned a human consciousness that does not know what is it like to be a bat into a bat’s consciousness that “knows” what is it like to be a bat. It seems that an idea of a human consciousness that has a structure of a bat’s consciousness is simply inconsistent because the identity of human consciousness depends of its having a structure of human consciousness (at least if we accept the fully structural account of consciousness defended in this paper).

It has been also argued that there is a fundamental and irreducible difference between objective and subjective knowledge about consciousness. I hope that the above presented ideas help also clarify the nature of that difference. Objective knowledge about some individual consciousness can be presented in structural terms and it is in fact a knowledge *about* a certain structure. In order to have such knowledge, one has to have access to all the relevant elements of that structure (in other words, one has to be related to that structure in a proper way). We may hope that one day the entire structure of consciousness will be discovered in some patterns of neural activity and that the community of neuroscientists will then have a chance to study it. A significant work toward that goal is already made. There are several theories besides the one of Crick and Koch, for example Varela (1999), Baars (1988), Dehaene et al. (1998), Lamme (2010), O’Brien and Opie (1999) and many others, that help us tracking and recognizing different structural features of consciousness in some neural activity patterns.

In order to have a subjective knowledge about some individual consciousness, on the other hand, one would have to *be* a cognitive system that *has* certain substructure of that individual consciousness. Subjective knowledge about certain consciousness is hence always a particular substructure of that very consciousness. We may say that if objective knowledge is in some sense an abstract phenomenon, then subjective knowledge is, according to the neurobiological view adopted in this paper, always some very concrete neural structure located in someone’s brain. We could in principle analyze and describe whichever individual instance of a certain subjective knowledge in perfectly objective and structural terms, but in order to actually *have* that subjective knowledge, we would have to, so to speak, turn a substructure of our own consciousness into the structure of that knowledge. So, the two concepts of knowledge, the objective and the subjective, are indeed different, and even a perfect objective epistemic access to the structure of a certain consciousness would not guarantee us the subjective knowledge about that consciousness. However, according to the framework presented here, this purely conceptual distinction does not imply any metaphysical distinctions or any philosophically problematic epistemic distinctions. Namely, it is easy to understand and accept that having knowledge about some neural structure does not necessarily make that structure occur in one’s brains.

## Conflict of Interest Statement

The author declares that the research was conducted in the absence of any commercial or financial relationships that could be construed as a potential conflict of interest.
